# ATP-sensitive potassium (K_ATP_) channel openers diazoxide and nicorandil lower intraocular pressure by activating the Erk1/2 signaling pathway

**DOI:** 10.1371/journal.pone.0179345

**Published:** 2017-06-08

**Authors:** Uttio Roy Chowdhury, Cindy K. Bahler, Bradley H. Holman, Michael P. Fautsch

**Affiliations:** Department of Ophthalmology, Mayo Clinic, Rochester, Minnesota, United States of America; Oregon Health and Science University, UNITED STATES

## Abstract

Elevated intraocular pressure is the most prevalent and only treatable risk factor for glaucoma, a degenerative disease of the optic nerve. While treatment options to slow disease progression are available, all current therapeutic and surgical treatments have unwanted side effects or limited efficacy, resulting in the need to identify new options. Previous reports from our laboratory have established a novel ocular hypotensive effect of ATP-sensitive potassium channel (K_ATP_) openers including diazoxide (DZ) and nicorandil (NCD). In the current study, we evaluated the role of Erk1/2 signaling pathway in K_ATP_ channel opener mediated reduction of intraocular pressure (IOP). Western blot analysis of DZ and NCD treated primary normal trabecular meshwork (NTM) cells, human TM (isolated from perfusion cultures of human anterior segments) and mouse eyes showed increased phosphorylation of Erk1/2 when compared to vehicle treated controls. DZ and NCD mediated pressure reduction (p<0.02) in human anterior segments (n = 7 for DZ, n = 4 for NCD) was abrogated by U0126 (DZ + U0126: -9.7 ± 11.5%, p = 0.11; NCD + U0126: -0.1 ± 11.5%, p = 1.0). In contrast, U0126 had no effect on latanoprostfree acid-induced pressure reduction (-52.5 ± 6.8%, n = 4, p = 0.001). In mice, DZ and NCD reduced IOP (DZ, 14.9 ± 3.8%, NCD, 16.9 ± 2.5%, n = 10, p<0.001), but the pressure reduction was inhibited by U0126 (DZ + U0126, 0.7 ± 3.0%; NCD + U0126, 0.9 ± 2.2%, n = 10, p>0.1). Histologic evaluation of transmission electron micrographs from DZ + U0126 and NCD + U0126 treated eyes revealed no observable morphological changes in the ultrastructure of the conventional outflow pathway. Taken together, the results indicate that the Erk1/2 pathway is necessary for IOP reduction by K_ATP_ channel openers DZ and NCD.

## Introduction

Glaucoma is a neurodegenerative disease affecting over 60 million people worldwide.[1] All current treatment strategies for clinical management of glaucoma are geared towards lowering IOP, the most prevalent and only treatable risk factor for the disease.[2] Commonly used glaucoma drugs lower IOP by either increasing aqueous humor removal from the anterior segment (e.g. prostaglandin analogs or cholinergic agents) or lowering aqueous humor secretion from the ciliary body (e.g. β-adrenergic blockers and carbonic anhydrase inhibitors).[3, 4] Unfortunately, all current glaucoma therapies have side effects and do not target the trabecular meshwork (TM) and Schlemm’s canal (SC), the primary tissues responsible for increased resistance to aqueous humor drainage through the conventional outflow pathway. None of these drugs directly protects the retina and the optic nerve, which are the primary tissues affected during glaucoma. As a result, development of new and improved glaucoma medications has been a priority for researchers worldwide.[5, 6]

ATP-sensitive potassium (K_ATP_) channels are hetero-octamers formed with 4 regulatory sulphonylurea receptor (SUR) subunits (SUR1, SUR2A or SUR2B) and 4 inwardly rectifying potassium channel subunits (K_ir_6.1 or K_ir_6.2).[7–9] These channels are found in tissues throughout the body including the cells and tissues of the conventional outflow pathway and the retina.[9–13] The opening and closing of K_ATP_ channels are regulated by changes in micro molar concentrations of intracellular ATP, connecting the energetic and metabolic states of the cells.[9, 14] K_ATP_ channels are involved in regulation of insulin secretion, glucose homeostasis and cellular stress adaptation.[8, 9, 15–17] Additionally, K_ATP_ channels have a role in cellular protection, particularly against damages caused by reactive oxidative species and ischemic injuries.[8, 9, 18, 19] As a result, the K_ATP_ channel openers are used to treat various cardiac pathologies.[20–23]

Our laboratory has established a novel ocular hypotensive property of several K_ATP_ channel openers.[9, 10, 13, 24] Our previous results indicate that SUR2B/K_ir_6.2 subunit containing K_ATP_ channels might be responsible for the ocular hypotensive activity of the K _ATP_ channel openers.[9, 13] However, the intracellular signaling mechanisms by which IOP reduction is achieved remain unknown. In non-ocular cells, K_ATP_ channel openers exert their physiological response through activation of the extracellular-signal regulated kinase 1/2 (Erk1/2).[25, 26] In light of this, we hypothesized that K_ATP_ channel openers lower IOP through activation of the Erk1/2 signaling pathway. To test this, we examined Erk1/2 phosphorylation, Erk1/2 inhibition and IOP in ex vivo, in vitro and in vivo model systems following treatment with the K_ATP_ channel opener’s diazoxide (DZ) and nicorandil (NCD).

## Methods

### Primary culture of human normal trabecular meshwork (NTM) cells

Three NTM cell lines (passage 3 to 7) derived from independent human donor eyes (age 3 months, 32 years and 57 years) were established as previously described.[27, 28] NTM cells were grown to confluence at 37^°^C in 5% CO_2_.

#### Study 1, evaluation of phosphorylated Erk1/2 protein

Confluent NTM cells were serum starved in DMEM containing antibiotics for 24 hours and treated with 20 μM DZ or NCD (Sigma-Aldrich, St. Louis, MO; diluted in serum-free DMEM from a 20 mM stock in DMSO). Concentration of 20 μM was chosen as it was the concentration that lowered pressure in previous human anterior segment perfusion culture studies.[10, 13, 24] After treatment, cells isolated at several time points between 15 min and 6 hours were lysed in ice cold lysis buffer (50 mM Tris, pH 8.0, 0.5% sodium dodecyl sulfate, 0.5% Triton X-100, 137 mM NaCl, 3 mM KCl, 8 mM Na_2_HPO_4_-7H_2_O, 1 mM KH_2_PO_4_) containing protease and phosphatase inhibitors (Roche, Indianapolis, IN). Total protein concentration was determined using Bradford’s assay.

#### Study 2, addition of U0126

To establish specificity of DZ action, primary NTM cells were pretreated with U0126 (Calbiochem, Billerica, MA; 20 μM, diluted in serum free DMEM from a 20 mM stock solution in DMSO), an Erk1/2 pathway inhibitor that targets MAPK/ERK (Mek) kinase, for 15 minutes followed by addition of DZ (20 μM) + U0126 (20 μM). Cells were lysed and total protein concentration was determined using Bradford’s assay.

#### Western blot

Cell lysates were mixed with 5X reducing sample buffer (Thermo Fisher Scientific, Waltham, MA) containing 15% 2-mercaptoethanol (Sigma-Aldrich). Total protein (12–15 μg) was loaded and separated on 4–15% gradient SDS-PAGE gels (Bio-Rad, Hercules, CA), transferred to polyvinylidene difluoride membranes (Millipore, Billerica, MA), and blocked in 2% non-fat dried milk as previously described.[27] Blocked membranes were probed with rabbit phospho Erk1/2 antibody (Cell Signaling Technology, Danvers, MA), total Erk1/2 antibody (Cell Signaling Technology) and horse-radish peroxidase conjugated anti-rabbit secondary antibody (GE Healthcare, Piscataway, NJ). Protein bands were visualized using ECL western blot signal detection reagent (GE Healthcare) and Kodak Biomax XAR films (Eastman Kodak, Rochester, NY). For total Erk1/2 detection, membranes were stripped with 5 M Guanidine hydrochloride (Sigma-Aldrich) prior to incubation with antibody. Films of phosphorylated Erk1/2 and total Erk1/2 were digitally scanned and analyzed with ImageJ software (developed by Wayne Rasband, National Institutes of Health, Bethesda, MD; available at http://rsb.info.nih.gov/ij/index.html). Phosphorylated Erk1/2 band intensity was determined following normalization to total Erk1/2 levels. The maximum band intensity across the time points was used to calculate the fold increase in comparison to corresponding normalized controls.

### Human anterior segment perfusion culture

Use of human donor eyes for this study was approved by the Mayo Clinic Institutional Review Board and adhered to the tenets of the Declaration of Helsinki. A total of 18 pairs of human eyes (age 73.8 ± 11.3 years, range 51 to 88 years) were used for this study. All eyes were obtained from the Minnesota Lions Eye Bank within 13.1 ± 2.6 hours of death. None of the donors had a documented history of eye disease and were not on any topical eye medications. For culture preparation, the eyes were bisected at the equator and the ciliary body, iris and lens were removed as previously described.[10, 24, 29–31] The resulting anterior segments were clamped in modified petri dishes and perfused with DMEM containing 1% antibiotic/antimycotic solution (Sigma-Aldrich) at the normal human aqueous humor flow rate of 2.5 μl/min. Anterior segment cultures were maintained at 37^°^C and pressure was recorded by a second cannula attached to a pressure transducer connected to a custom designed software system that recorded hourly pressure readings by averaging 60 one-minute pressure measurements. Outflow facility was calculated at 0 hour (C_0_) and 24 hours (C_d_) following drug treatments by dividing the flow rate by pressure at respective time points.

#### Study 1, evaluation of phosphorylated Erk1/2

In 3 pairs of human eyes (age 49, 81 and 81 years), one anterior segment of each pair was perfused with 20 μM DZ (diluted with DMEM from a 20 mM stock in DMSO) while the contralateral anterior segment was perfused with vehicle for 6 hours (n = 1) and 14 hours (n = 2). TM tissue was dissected from these anterior segments and homogenized individually in 120 μl lysis buffer with protease and phosphatase inhibitors (described above) [27, 32] using an ultrasonic cell disruptor (Misonix, Farmingdale, NY). Protein concentration was determined by Bradford assay. On average, TM tissue from each eye yielded 0.60 ± 0.09 μg/μl (n = 3) total protein. Western blot for phosphorylated Erk1/2 and total Erk1/2 was performed with 15 μg total protein per lane, as described above.

#### Study 2, treatment with DZ and DZ + U0126

In 7 pairs of eyes (age 76.4 ± 12.3 years, range 54 to 86 years), one anterior segment was perfused for 24 hours with 20 μM DZ while the contralateral anterior segment received vehicle.[10] After perfusion of DZ alone, DZ (20 μM) + U0126 (20 μM; prepared from a 20 mM stock by diluting with DMEM) was perfused for an additional 24 hours. Contralateral eye received appropriate vehicle.

#### Study 3, treatment with NCD and NCD + U0126

In 4 pairs of eyes (age 62.5 ± 9.7 years, range 51 to 71 years) one anterior segment received NCD (20 μM; prepared as described for DZ) for 24 hours while the contralateral eye received vehicle. Following NCD treatment, 3 out of the 4 anterior segments that received NCD only, were treated with NCD (20 μM) + U0126 (20 μM) for an additional 24 hours while contralateral eyes received appropriate vehicle.

#### Study 4, treatment with DZ, LFA and LFA + U0126

In 4 pairs of eyes (age 79.0 ± 7.8 years, range 69 to 88 years) one anterior segment from each pair received 20 μM DZ for 24 hours followed by DZ (20 μM) + U0126 (0.5 mM) for an additional 24 hours. Subsequently, LFA (0.1 μM) + U0126 (0.5 mM) was added for another 24 hours. Contralateral eye was treated with appropriate vehicle at designated drug treatment times. Final LFA (Cayman Chemical, Ann Arbor, MI) concentration was prepared by diluting a 100 mM stock dissolved in ethanol with DMEM.

#### Study 5, treatment with U0126 alone

In 7 pairs of eyes (age 68.3 ± 11.0 years; range 51 to 84 years), 0.5 mM U0126 was added to one anterior segment while the fellow eye received vehicle.

### Animals

Use of animals and experimental protocols were pre-approved by the Mayo Clinic Institutional Animal Care and Use Committee (IACUC) and adhered to the tenets of the ARVO Statement for the Use of Animals in Ophthalmic and Vision Research. Wild type C57BL/6 mice (retired breeders, age >8 months) were purchased from Charles River Laboratories (Wilmington, MA) and maintained at the Mayo Clinic animal care facility under a 12 hour light and dark cycle. Animals received standard rodent chow and water *ad libitum*. Animals were acclimated to their new environment for ≥5 days before initiation of an experiment.

#### Study 1, detection of phosphorylated Erk1/2

One eye of 6 mice were treated with 5 mM DZ [(prepared by diluting a 100 mM stock (in DMSO) in 10% polyethoxylated castor oil (Cremophor EL; Sigma-Aldrich); delivered as a 5 μl bolus, equivalent to 25 nmol)] while the contralateral eye received vehicle. Following 15 minute treatment, animals were euthanized by CO_2_ asphyxiation, eyes were enucleated, and micro-dissection was performed to remove the cornea along with the TM and SC from each animal. Anterior segment tissues from all 6 animals were pooled into appropriate treatment and control groups, incubated in cell lysis buffer (described above for western blots) and homogenized using an ultrasonic cell disruptor (Misonix). Total protein was assayed by Bradford’s method. Western blot for phosphorylated Erk1/2 and total Erk1/2 was performed as described above.

#### Study 2, treatment with DZ, NCD, DZ + U0126 and NCD + U0126

Prior to addition of drugs, baseline IOP was measured 3 times daily for 3 consecutive days with a handheld rebound tonometer (Icare Tonolab: Colonial Medical Supply, Franconia, NH) in live non-anesthetized mice as previously described.[13, 24] Daily IOP was determined by taking the average of the 3 independent time points. At the end of pretreatment, one eye of each mouse was treated with either 25 nmol DZ or 25 nmol NCD (n = 10 for each drug; delivered topically as a 5 μl bolus) daily for 5 consecutive days while the contralateral eye received vehicle daily during the same period. IOP was recorded daily at 1, 4 and 23 hours following treatment. At the completion of the 5 day treatment period, eyes receiving DZ or NCD were treated with DZ (25 nmol) + U0126 (2.5 nmol) or NCD (25 nmol) + U0126 (2.5 nmol) for another 5 days. U0126 was prepared from a 20 mM DMSO stock in the same solutions containing the working concentrations of DZ and NCD. On completion of treatment, 4 mice were sacrificed from DZ + U0126 and NCD + U0126 treatment groups, eyes were enucleated and placed in 10% neutral buffered formalin. With the remaining animals (n = 6 per group), drug treatment was stopped but IOP was recorded and monitored at 3 time points corresponding to those used during treatment, for 3 consecutive days.

### Histology

#### Human anterior segments

At the end of each study, two tissue wedges containing the TM and SC were dissected 180° apart from each anterior segment and fixed in 10% neutral buffered formalin. Tissue wedges were post fixed in 2% osmium tetroxide (Electron Microscopy Sciences, Hatfield, PA) in 0.1 M phosphate buffer followed by dehydration in ascending ethanol concentrations. Tissues were subjected to a clearing agent (acetone, Sigma-Aldrich), embedded in epoxy resin blocks, and 500 nm and 100 nm sections were obtained using an ultramicrotome (Leica Microsystems, Buffalo Grove, IL). 500 nm sections were stained with toluidine blue and gross morphology was assessed by light microscopy. 100 nm sections were placed on copper grids and stained with 2% uranyl acetate (Electron Microscopy Sciences) followed by lead citrate (Mager Scientific, Dexter, MI). Sections were imaged using a JEOL 1400 transmission electron microscope (JEOL USA, Peabody, MA) for evaluation of cell and tissue ultrastructure.

#### Mouse eyes

Enucleated mouse eyes were placed in 10% neutral buffered formalin for at least 24 hours. Whole eyes were processed as described for human anterior segments. Whole eyes were cut longitudinally and 500 nm and 100 nm sections were used for toluidine blue staining and transmission electron microscopy as described above.

#### Statistics

All IOP data are represented as absolute IOP or as absolute change compared to control (ΔIOP in mmHg) and are obtained by subtracting the average IOP of the treated eye from that of the control eye at any given time point. Values are represented as mean ± standard deviation. Data between control and treated groups were compared using Student’s paired t test. Differences were considered significant when p≤0.05.

## Results

### DZ and NCD treatment increases Erk1/2 phosphorylation

**Fig 1:**
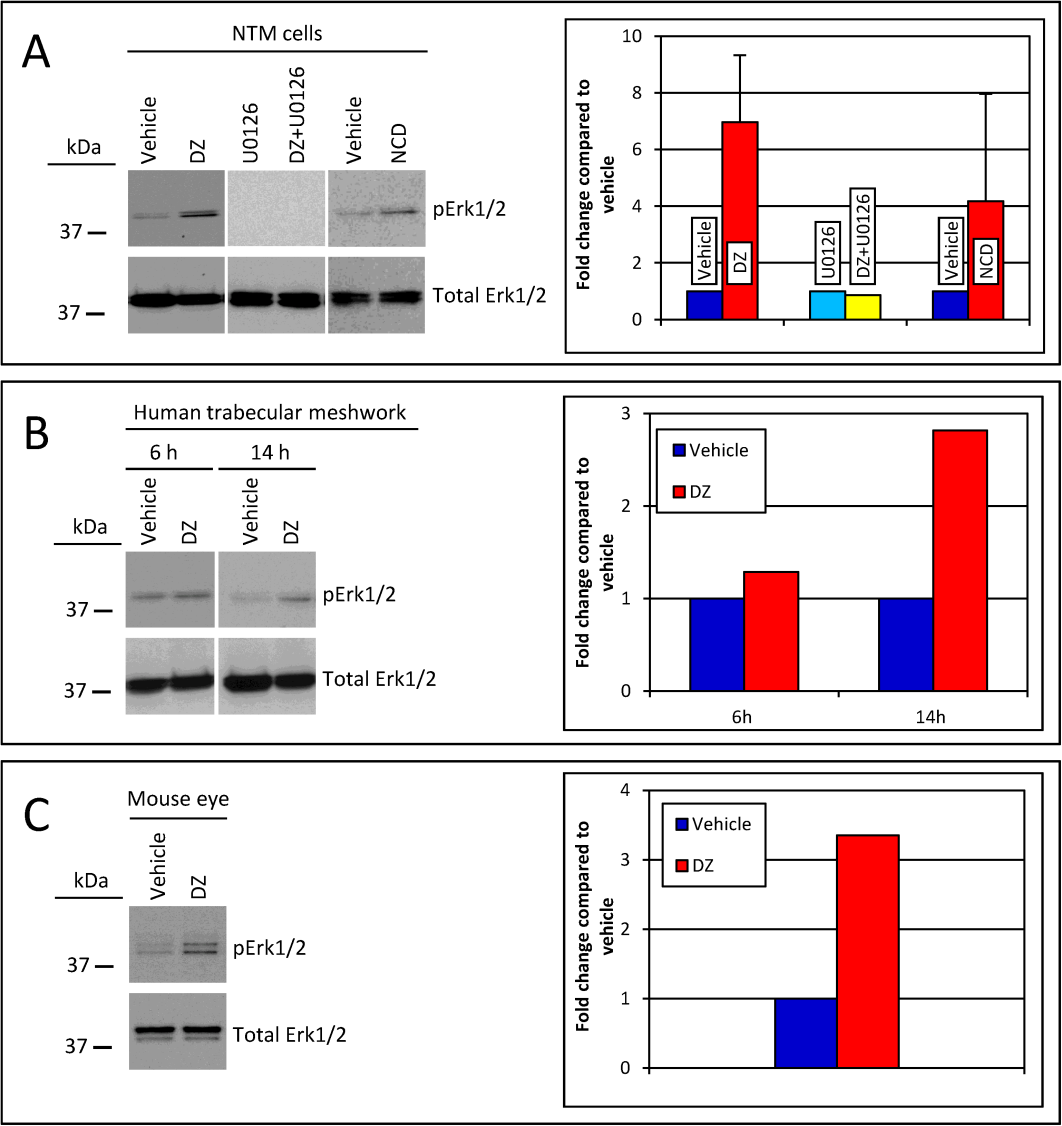
Treatment with DZ and NCD increases Erk1/2 phosphorylation. (A) DZ and NCD caused upregulation of Erk1/2 phosphorylation in NTM cells (n = 2) within 6 hours of treatment. Phosphorylation of Erk1/2 was eliminated by the Erk1/2 pathway inhibitor U0126 either by itself or in the presence of DZ. (B) Human anterior segments treated with DZ for 6 h (n = 1) showed minimal change in phosphorylated Erk1/2. However, following 14 hours (n = 2) of DZ treatment, an increase in Erk1/2 phosphorylation was observed. (C) In vivo topical application of DZ to mouse eyes caused upregulation of Erk1/2 phosphorylation within 15 minutes of treatment. Mouse anterior segments from 6 DZ treated eyes and 6 vehicle treated eyes were pooled for this experiment. pErk1/2, phosphorylated Erk1/2.

To evaluate the effect of DZ and NCD on phosphorylated Erk1/2, we treated primary cultures of human NTM cells (in vitro), anterior human segments (ex vivo), and mouse eyes (in vivo) with DZ and NCD. In primary NTM cells, elevated levels of phosphorylated Erk1/2 were identified within 6 hours of treatment with DZ (7.0 ± 2.4 fold; n = 2) and NCD (4.2 ± 3.8 fold; n = 2) (Fig 1A). When NTM cells were treated with U0126, an inhibitor of the Erk1/2 pathway, either by themselves or in combination with DZ, no increase in phosphorylated Erk1/2 was observed. In human eyes perfused with DZ or vehicle for 6 or 14 hours, protein lysates isolated from TM cells showed a nominal increase in phosphorylated Erk1/2 at 6 hours, but a 2.9 ± 0.2 fold change was observed in phosphorylated Erk1/2 by 14 hours (n = 2; Fig 1B). Likewise, protein lysates of anterior segments isolated from wild type C57BL/6 mice (n = 6, pooled lysates) treated with DZ showed increased expression of phosphorylated Erk1/2 within 15 minutes of topical eye administration compared to vehicle treated contralateral eyes. (Fig 1C)

### Erk1/2 activation is necessary for DZ and NCD mediated pressure reduction

**Fig 2:**
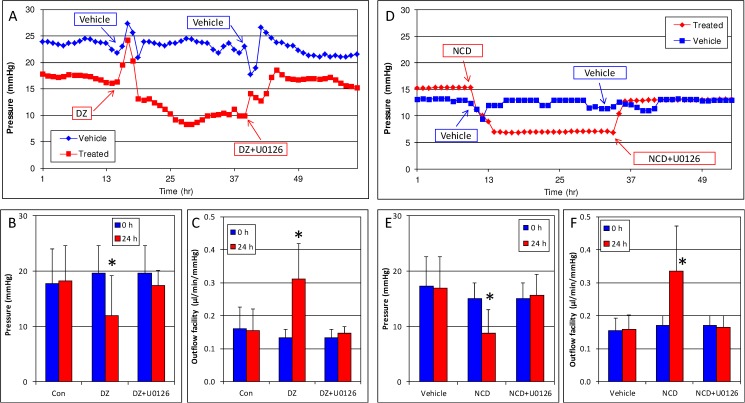
DZ and NCD lower pressure by activating the Erk1/2 pathway in human anterior segments. (A-C) Addition of DZ caused significant reduction of pressure (A, B) and increase in outflow facility (C). However, addition of the Erk1/2 pathway inhibitor U0126 completely inhibited the pressure reduction (A, B) and outflow facility increase (C) caused by DZ. (D-F) Similar to results obtained with DZ, U0126 also inhibited the ocular hypotensive effects of NCD (D, E). Outflow facility increases caused by NCD treatment were reversed by U0126 (F). Graphs are representative images, *p<0.05.

To determine whether the Erk1/2 pathway was the main signaling axis through which DZ and NCD lowered pressure, we evaluated DZ in the presence of U0126 in the human anterior segment perfusion culture model. Similar to a previously published report,[10] human anterior segments (n = 7) showed significant reduction of pressure over a 24 hour period following perfusion with 20 μM DZ (19.7 ± 4.9 mmHg at 0 hour vs. 12.0 ± 7.2 mmHg at 24 hours; p<0.001) compared to vehicle treated eyes (17.7 ± 6.3 mmHg at 0 hour vs. 18.3 ± 6.3 mmHg at 24 hours, p = 0.28). When U0126 (20 μM) was added in the presence of DZ, pressure reduction was eliminated and pressure returned to baseline levels within 24 hours (baseline, 19.7 ± 4.9 mmHg; DZ + U0126, 17.4 ± 2.7 mmHg, p = 0.11) (Fig 2A and 2B). Correspondingly, outflow facility increased with DZ treatment alone (0.13 ± 0.03 μl/min/mmHg at 0 hour vs. 0.27 ± 0.14 μl/min/mmHg at 24 hours; p = 0.02) but returned to baseline levels following DZ + U0126 combination treatment for 24 hours (baseline, 0.13 ± 0.03; DZ + U0126, 0.15 ± 0.02 μl/min/mmHg p = 0.06) (Fig 2C, Table 1). Vehicle showed no effect (0.16 ± 0.07 μl/min/mmHg at 0 hour vs. 0.16 ± 0.07 μl/min/mmHg at 24 hours, p = 0.88) throughout the experiment.

**Table 1:** Effect of treatments on outflow facility in perfusion cultured human anterior segments.

Study number	Treatment	n	0 h (μl/min/mmHg)	24 h (μl/min/mmHg)
	DZ	7	0.13 ± 0.03	0.27 ± 0.14*
	DZ+U0126	7	0.13 ± 0.03	0.15 ± 0.02
	Vehicle	7	0.16 ± 0.07	0.16 ± 0.07
2	NCD	4	0.17 ± 0.03	0.33 ± 0.14*
	NCD+U0126	3	0.17 ± 0.03	0.17 ± 0.04
	Vehicle	4	0.15 ± 0.04	0.16 ± 0.04
3	U0126	7	0.18 ± 0.03	0.18 ± 0.03
	Vehicle	7	0.17 ± 0.04	0.15 ± 0.03
4	DZ	4	0.14 ± 0.03	0.38 ± 0.14*
	DZ+U0126	4	0.14 ± 0.03	0.12 ± 0.03
	LFA + U0126	4	0.13 ± 0.03	0.30 ± 0.19*
	Vehicle	4	0.25 ± 0.06	0.29 ± 0.09

* p<0.05

To verify results obtained with DZ, we investigated the effect of NCD, a separate K_ATP_ channel opener, on pressure and outflow facility of cultured human anterior segments. When anterior segments (n = 4) were treated with NCD alone, pressure was reduced from 15.0 ± 2.8 mmHg at 0 hour to 8.8 ± 4.4 mmHg at 24 hours (p = 0.01) (Fig 2D and 2E). No effect was observed in vehicle treated eyes (17.3 ± 5.3 mmHg at 0 hour vs. 17.0 ± 5.6 mmHg at 24 hours, p = 0.39). When U0126 was added to three of the four anterior segments in the presence of NCD, pressure returned to baseline levels (baseline, 15.7 ± 3.1 mmHg; NCD + U0126, 15.7 ± 3.8 mmHg at 24 hours, p = 1.0, n = 3) (Fig 2D and 2E). Outflow facility changes with NCD corresponded to a 96.6% increase in 24 hours (0.17 ± 0.03 μl/min/mmHg at 0 hour vs. 0.33 ± 0.14 μl/min/mmHg at 24 hours, p = 0.03) (Fig 2F, Table 1). With the addition of U0126, outflow facility returned to baseline within 24 hours (0.17 ± 0.04 μl/min/mmHg, p = 0.0.9).(Fig 2F, Table 1).

**Fig 3:**
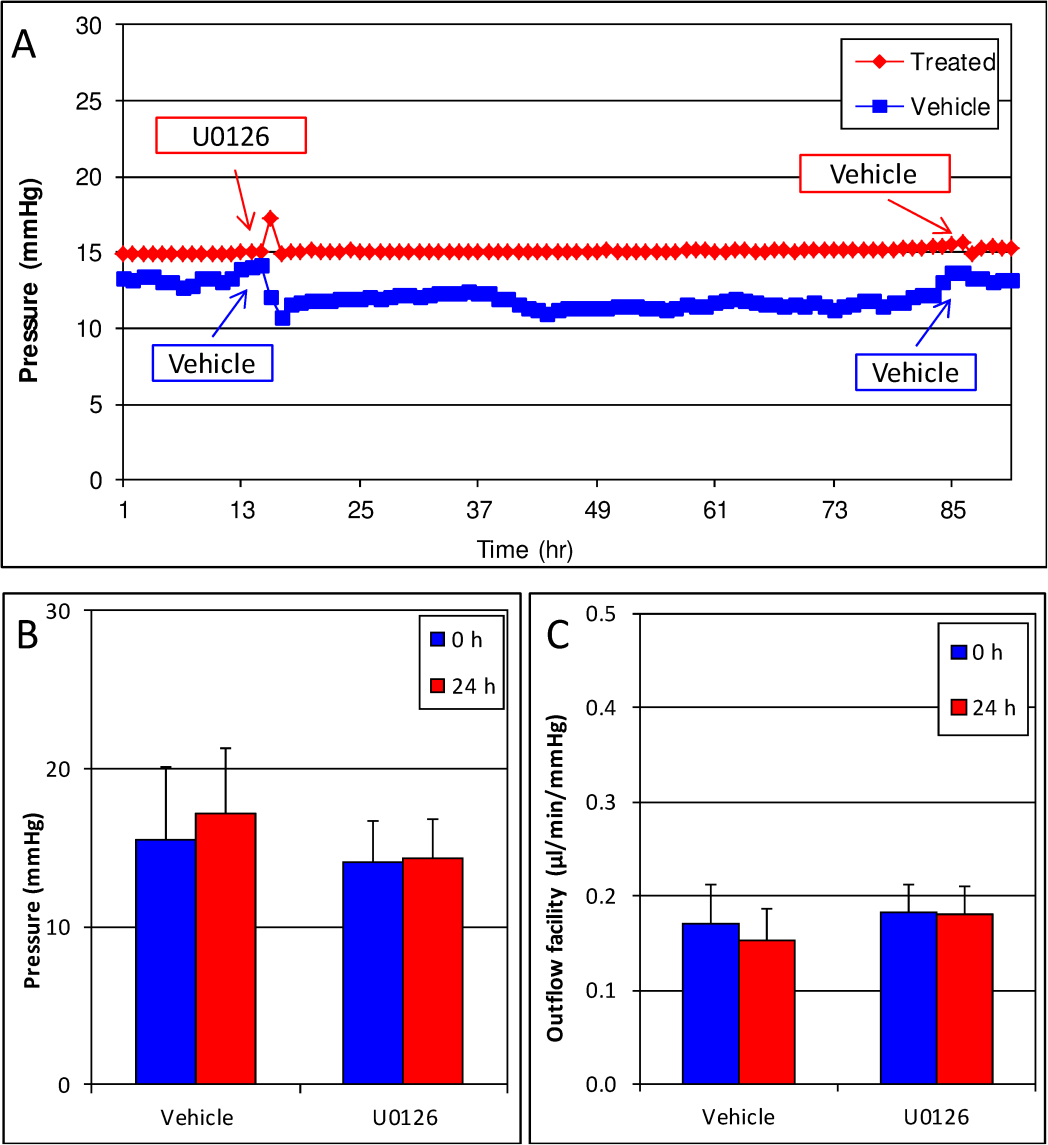
U0126 does not affect pressure or outflow facility by itself. Human anterior segments were treated with the Erk1/2 pathway inhibitor U0126. U0126 or vehicle treatment had no effect on pressure (A, B) and outflow facility (C). Graph is representative of experiments performed in 7 separate eye pairs.

To determine if Erk1/2 was essential for IOP, we treated human anterior segments with U0126 alone. No change in pressure (vehicle 15.6 ± 4.6 mmHg at 0 hour and 17.1 ± 4.1 mmHg at 24 hours, p = 0.17; U0126, 14.1 ± 2.6 mmHg at 0 h and 14.3 ± 2.6 mmHg at 24 hours, p = 0.69) or outflow facility (vehicle, 0.17 ± 0.04 μl/min/mmHg at 0 hour to 0.15 ± 0.03 μl/min/mmHg at 24 hours; U0126, 0.18 ± 0.03 μl/min/mmHg at 0 hour and 0.18 ± 0.03 at 24 hours, p = 0.72) was noted in these eyes (Fig 3A–3C, Table 1).

**Fig 4:**
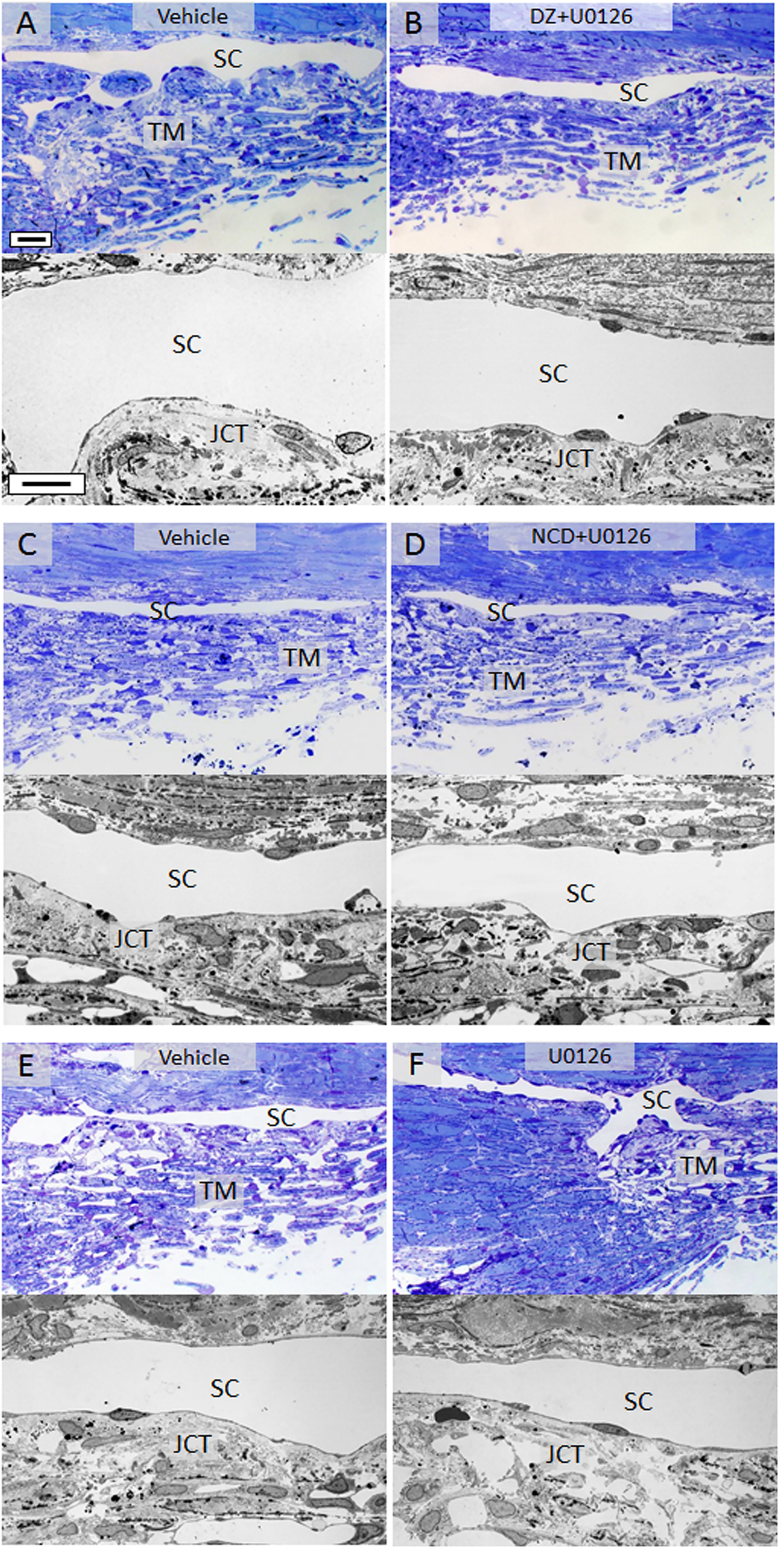
Morphology of JCT and SC following DZ and NCD treatment with and without U0126. Cells and tissues of the conventional outflow pathway were histologically evaluated from toluidine blue stained sections and transmission electron micrographs of eyes treated with vehicle (A) or DZ and DZ + U0126 (B), vehicle (C) or NCD and NCD + U0126 (D) and vehicle (E) or U0126 by itself (F). Overall, all micrographs showed intact trabecular beams traversed by viable trabecular meshwork cells. Extracellular matrix deposition in the juxtacanalicular region was similar to that observed in corresponding vehicle controls. Schlemm’s canal inner and outer walls were also intact in control and treated groups. Representative images are shown. Scale bar, 20 μm for toluidine blue sections; 10 μm for transmission electron microscopy. TM, trabecular meshwork; SC, Schlemm’s canal; JCT, juxtacanalicular tissue.

To assess the effect of DZ, NCD and U0126 on conventional outflow morphology, we sectioned various tissue wedges isolated from treated anterior segments and processed them for toluidine blue staining and transmission electron microscopy. Treatment with DZ, NCD and U0126, in various combinations did not cause detrimental side effects to the overall morphology and the ultrastructure of the cells in the TM and inner and outer walls of SC in comparison to vehicle treated controls (Fig 4A–4F). No specific changes in extracellular matrix ultrastructure were noted during these acute treatments.

### Erk1/2 signaling is required for DZ and NCD mediated intraocular pressure reduction in C57BL/6 mice

**Fig 5:**
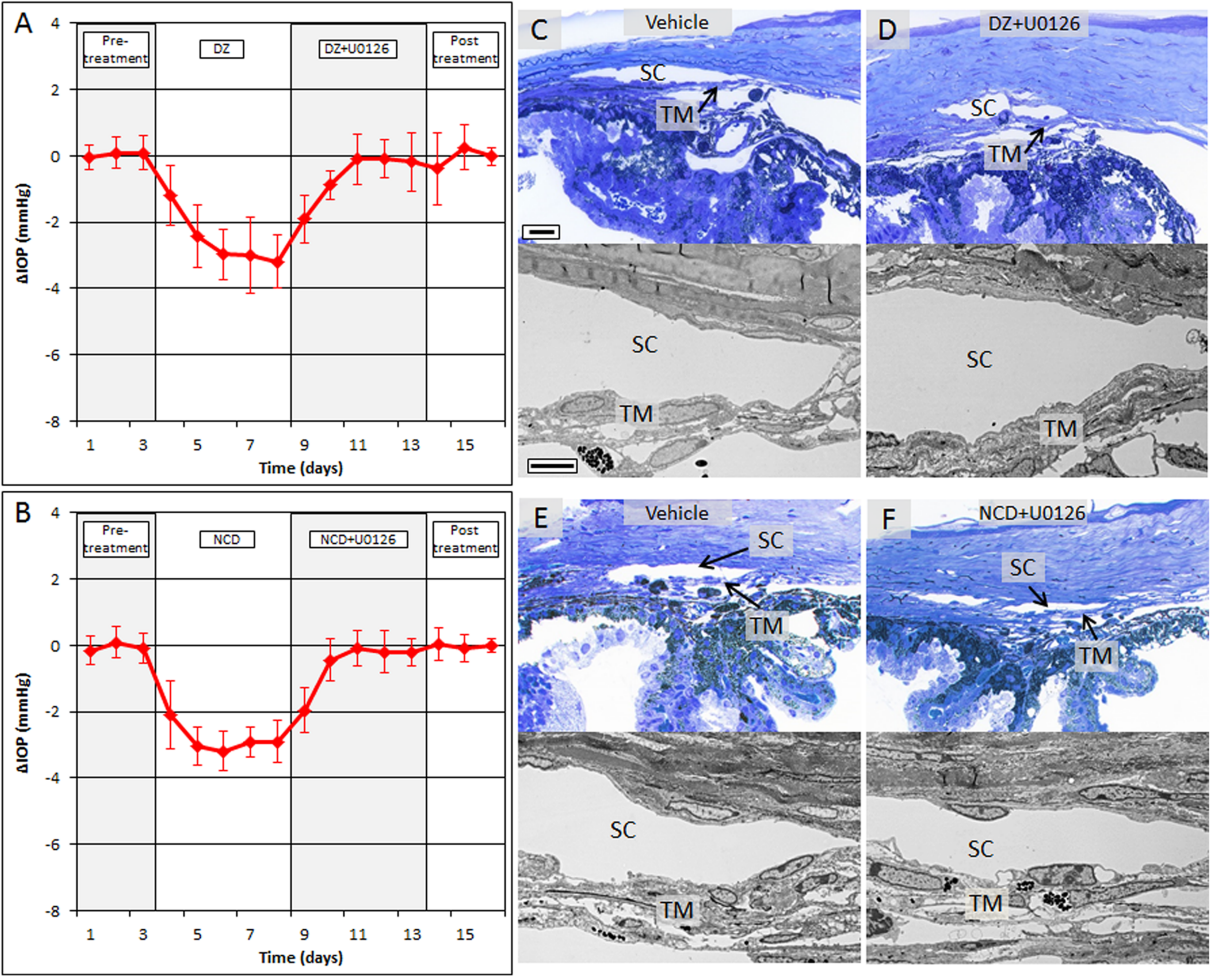
Ocular hypotensive effects of DZ and NCD were inhibited by U0126 *in vivo*. (A) Mice treated with topical DZ eye drops showed an average IOP reduction of 14.9 ± 3.8% (n = 10, p<0.001) which was inhibited by addition of U0126. IOP returned to baseline after three days of DZ + U0126 treatment (vehicle control, 17.5 ± 0.5 mmHg; DZ + U0126, 17.4 ± 0.7 mmHg; n = 10, p = 0.7). (B) Similar to DZ treatment, U0126 treatment in the presence of the ocular hypotensive agent NCD returned IOP to baseline values within 3 days. (C-F) Assessment of the conventional outflow pathway gross morphology and tissue ultrastructure following treatment with vehicle (C) and DZ + U0126 (D) or vehicle (E) and NCD + U0126 (F) showed healthy living cells in the trabecular meshwork with an open and intact Schlemm’s canal. Scale bar, 20 μm for toluidine blue sections; 5 μm for TEM. SC, Schlemm’s canal; TM, trabecular meshwork.

To evaluate the association of IOP reduction by K_ATP_ channel openers and Erk1/2 phosphorylation in vivo, C57Bl/6 mice were treated with DZ (25 nmol) and NCD (25 nmol) in the presence or absence of U0126 (2.5 nmol). DZ lowered IOP by 2.6 ± 0.7 mmHg (range of 1.2 ± 0.9 mmHg to 3.2 ± 0.8) which resulted in a 14.9 ± 3.8% decrease in IOP compared to fellow vehicle treated eyes (Fig 5A). Addition of U0126 with DZ eliminated the IOP reduction caused by DZ alone, returning IOP back to baseline levels after three days of treatment with DZ + U0126 (vehicle control, 17.5 ± 0.5 mmHg; DZ + U0126, 17.4 ± 0.7 mmHg; n = 10, p = 0.7). Similar results were obtained with NCD. Treatment with NCD alone lowered pressure by 2.8 ± 0.4 mmHg (range 2.1 ± 1.0 to 3.2 ± 0.6 mmHg), a 16.9 ± 2.5% change compared to fellow vehicle treated control eyes (Fig 5B). Addition of U0126 inhibited NCD’s IOP reduction, returning IOP to baseline levels by the end of the third day of treatment (vehicle control, 16.7 ± 0.5 mmHg; NCD + U0126, 16.6 ± 0.7 mmHg; n = 10, p = 0.61). Evaluation of toluidine blue stained thin sections and transmission electron micrographs of the conventional outflow pathway in the DZ and NCD treated mice did not show any ultrastructural damage to the cells and tissues (Fig 5C–5F). TM from both control and treated groups contained healthy and viable cells as noted from the shape and structure of the nuclei along with the presence of homogenous cytoplasm around the cells. The cells were regularly interspersed on the TM beams and there were no apparent signs of any kind of cellular stress or deformity as a side effect of treatment. SC appeared open with intact inner and outer walls. Overall, no toxic effects of DZ, NCD and U0126 were observed.

### Pressure reduction by LFA was not affected by U0126 in human anterior segment perfusion cultures

**Fig 6:**
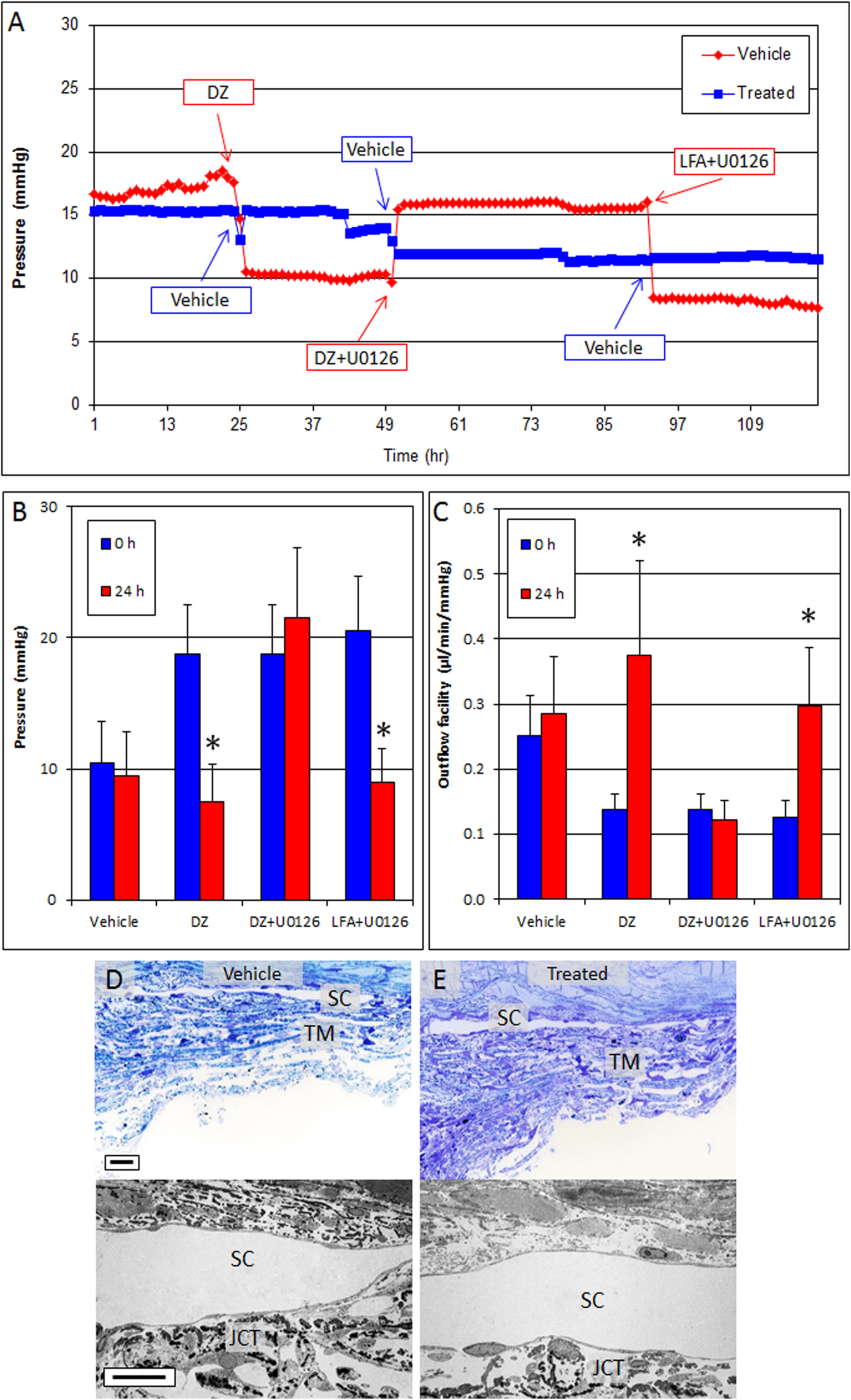
U0126 inhibits ocular hypotensive effects of DZ but not of LFA in perfusion cultured human anterior segments. (A-C) Anterior segments treated with DZ showed decreased pressure (A, B) and increased outflow facility (C) which returned to baseline following treatment with DZ + U0126. In contrast, U0126 was unable to inhibit pressure reduction caused by LFA, indicating that utilization of the Erk1/2 pathway for lowering IOP was different between DZ and LFA. (D and E) Evaluation of histology using toluidine blue sections and transmission electron micrographs showed regular extracellular matrix deposition in JCT and intact trabecular meshwork beams and viable cells in the outflow pathway of vehicle (D) and treated (E) eyes. Representative micrographs are shown. Scale bar, 20 μm for toluidine blue sections; 10 μm for TEM; *p<0.05; TM, trabecular meshwork; SC, Schlemm’s canal; JCT, juxtacanalicular tissue.

To determine whether the Erk1/2 pathway was unique to K_ATP_ channel openers or was a more generalized pathway utilized in pressure reduction, we evaluated the effect of U0126 on anterior segments (n = 4) treated with DZ and the prostaglandin analog LFA. Anterior segments treated with DZ alone reduced pressure from 18.8 ± 3.8 mmHg at 0 hour to 7.5 ± 2.9 mmHg at 24 hours (p = 0.002). This pressure reduction was inhibited by addition of U0126 (baseline, 18.8 ± 3.8 mmHg; DZ + U0126, 21.5 ± 5.3 mmHg; p = 0.37) (Fig 6A and 6B). In contrast, in the same eyes, U0126 was not able to inhibit LFA from reducing pressure (baseline, 20.5 ± 4.2 mmHg; LFA + U0126, 9.0 ± 2.6 mmHg; p = 0.01). On average, outflow facility was increased by 170% with DZ treatment (0.14 ± 0.03 μl/min/mmHg at 0 hour and 0.38 ± 0.14 μl/min/mmHg at 24 hours, p = 0.04), by -17% with DZ + U0126 (baseline, 0.14 ± 0.03 μl/min/mmHg; DZ + U0126, 0.12 ± 0.03; p = 0.46), and by 110% with LFA + U0126 (baseline, 0.14 ± 0.03 μl/min/mmHg; LFA + U0126, 0.30 ± 0.09 μl/min/mmHg, p = 0.04) (Fig 6C, Table 1). Histologic evaluation of toluidine blue sections and transmission electron micrographs of the conventional outflow pathway revealed healthy cell and tissue morphology following treatment (Fig 6D and 6E). Inner and outer walls of SC were found to be intact in both treated and control eyes.

## Discussion

Our previous studies have elucidated a novel ocular hypotensive property of several K_ATP_ channel openers, including DZ and NCD.[9, 10, 13, 24] Both DZ and NCD lower IOP by opening K_ATP_ channels containing SUR2B/K_ir_6.2 subunits and possibly targeting underlying physiologic pathways.[9, 10, 13, 24] However, the exact intracellular signaling pathway associated with IOP reduction following K_ATP_ channel opening were unknown until now. In the current study, we identified that opening of K_ATP_ channels by addition of DZ or NCD leads to enhanced phosphorylation of Erk1/2 and that Erk1/2 activation is required for their ability to reduce IOP. These results suggest that IOP lowering effects of DZ and NCD are mediated through the Erk1/2 signaling pathway.

The MAP kinase pathway is a highly conserved mammalian second messenger system. Of the various MAP kinase pathways, the Ras-Raf-Mek-Erk pathway (also known as the Erk1/2 or p42/p44 MAP kinase pathway) is the best characterized.[33, 34] Depending on the type of ligand and context of activation, the Erk1/2 pathway can regulate a large number of vital functions including cell growth and proliferation, transcriptional regulation and metabolism.[33] In ocular cells, the Erk1/2 pathway has been shown to be involved in activation of matrix metalloproteinases and TM extracellular matrix turnover leading to IOP regulation.[35, 36] U0126 is a highly selective inhibitor (IC50, 0.5 μM) of the Erk1/2 pathway. [37] It inhibits both active and inactive forms of Mek1/2, a kinase whose only downstream targets are Erk1/2. Mek1/2 is the chief regulatory step of the Erk1/2 signaling pathway and all endogenous activators of the Erk signaling pathway targets Mek1/2.[38–40] Because of this, U0126, a highly selective inhibitor of Mek1/2, is an efficient and commonly utilized agent for studying physiologic roles of the Erk1/2 signaling pathway.[41, 42]

Various kinds of cellular insults (e.g. hypoxia, reactive oxygen species and oxidative stress, etc.) that have been linked to glaucoma [43–45] are known to affect the Erk1/2 pathway.[46] Moreover, surgical methods for glaucoma treatment like trabeculectomies are thought to aid in normalization of TM tissue by stimulating secretion of TNFα and restoring TM extracellular matrix through the Erk1/2 pathway.[35] Erk1/2 has also been shown to be directly upregulated as a cellular response to increased IOP[46] in a rabbit[47] and a rat model[48] of elevated IOP. Based on these studies, it has been hypothesized that Erk1/2 could be an important target for glaucoma therapeutics.[46] In the current study, eyes treated with the Erk1/2 pathway blocker U0126 inhibited IOP reduction following K_ATP_ channel opening with DZ and NCD, suggesting that downstream effects of these compounds are directly mediated through the Erk1/2 signaling pathway. This mode of Erk activation seems to be unique and specific to the K_ATP_ channel openers DZ and NCD, as intraocular pressure reduction caused by DZ and NCD treatment was eliminated by co-treatment with the Erk1/2 inhibitor U0126. In contrast, U0126 added to eyes treated with LFA did not affect its intraocular pressure reduction capabilities, indicating that LFA utilizes a separate pathway for inducing its ocular hypotensive effects. Activating two separate pathways by using one K_ATP_ channel opener and a prostaglandin analog may explain the additive effect of these drugs in IOP reduction as previously reported by our laboratory using LFA and the K_ATP_ channel opener cromakalim.[24] The ability to influence a key endogenous signaling pathway that is involved in regulation of intraocular pressure makes DZ and NCD a unique class of ocular hypotensive agents.

One of the hallmark pathological changes in glaucoma is the damage to retinal ganglion cells.[49] In retinal ganglion cells with axonal injury, pro-apoptotic factors Bim and Bax are activated leading to initiation of the apoptotic cascade. [50–52] One of the ways by which the Erk1/2 pathway protects cells from apoptotic events is by degrading Bim through phosphorylation of specific serine residues.[53, 54] It is tempting to speculate that activating the Erk1/2 pathway through the use of agents like K_ATP_ channel openers may be a means to protecting retinal ganglion cells during glaucoma. Studies have shown that several retinal neuroprotective agents like pituitary adenylate cyclase activating polypeptide (PACAP), insulin-like growth factor-1 (IGF-1) and GM-CSF use the Erk1/2 pathway to promote retinal ganglion cell survival.[55–59] Additionally, several studies have shown that the K_ATP_ channel opener DZ has a direct neuroprotective effect on retinal ganglion cells during events of ischemia or glutamate induced cellular insults.[60–62] However, it remains to be evaluated whether the neuroprotective role of DZ is orchestrated through Erk mediated downregulation of pro-apoptotic proteins like Bim and Bax in the retinal ganglion cells.

The intraocular pressure reduction that we observed in human and mouse eyes following treatment with DZ and NCD was similar to that noted in our previous reports.[10, 13] This helps to validate the efficacy of the murine models for the current experiments. However, the use of normotensive mice can be considered a limitation for a study that is trying to elucidate physiology of a disease that occurs in humans. Nevertheless, data have established that anatomy of mouse eyes is similar to humans and normotensive mice have also been used to study the efficacy of clinically used glaucoma drugs.[63–65] Our previous reports have also established the usefulness of this model in studying the IOP lowering properties of K_ATP_ channel openers.[10, 13, 24]

In summary, data from the current study elucidates that the K_ATP_ channel openers DZ and NCD lower IOP by specifically activating the Erk1/2 signaling pathway in relevant ocular cells. The Erk1/2 signaling axis is one of the single most important pathways involved in survival and proliferation of various cells. The fact that K_ATP_ channel openers can target the Erk1/2 signaling pathway suggests these molecules may be candidate therapeutic agents for management of ocular hypertensive diseases like glaucoma.

## Supporting information

S1 File ARRIVE guideline checklist-DZ-ERK. (PDF)
